# Comparative Genomics of *Escherichia coli* Isolated from Skin and Soft Tissue and Other Extraintestinal Infections

**DOI:** 10.1128/mBio.01070-17

**Published:** 2017-08-15

**Authors:** Amit Ranjan, Sabiha Shaik, Nishant Nandanwar, Arif Hussain, Sumeet K. Tiwari, Torsten Semmler, Savita Jadhav, Lothar H. Wieler, Munirul Alam, Rita R. Colwell, Niyaz Ahmed

**Affiliations:** a Pathogen Biology Laboratory, Department of Biotechnology and Bioinformatics, University of Hyderabad, Gachibowli, Hyderabad, India; b Centre for Infection Medicine, Institute of Microbiology and Epizootics, Freie Universität Berlin, Berlin, Germany; c Department of Microbiology, Dr. D. Y. Patil Medical College, Hospital and Research Centre Pimpri (Dr. D. Y. Patil Vidyapeeth), Pune, India; d Robert Koch Institute, Berlin, Germany; e International Centre for Diarrheal Disease Research, Bangladesh (icddr,b), Mohakhali, Dhaka, Bangladesh; f Maryland Pathogen Research Institute, University of Maryland, College Park, Maryland, USA; g University of Maryland Institute of Advanced Computer Studies, University of Maryland, College Park, Maryland, USA; h Johns Hopkins Bloomberg School of Public Health, Baltimore, Maryland, USA; Institut Pasteur

**Keywords:** *Escherichia coli*, genomics, sepsis

## Abstract

*Escherichia coli*, an intestinal Gram-negative bacterium, has been shown to be associated with a variety of diseases in addition to intestinal infections, such as urinary tract infections (UTIs), meningitis in neonates, septicemia, skin and soft tissue infections (SSTIs), and colisepticemia. Thus, for nonintestinal infections, it is categorized as extraintestinal pathogenic *E. coli* (ExPEC). It is also an opportunistic pathogen, causing cross infections, notably as an agent of zoonotic diseases. However, comparative genomic data providing functional and genetic coordinates for ExPEC strains associated with these different types of infections have not proven conclusive. In the study reported here, ExPEC *E. coli* isolated from SSTIs was characterized, including virulence and drug resistance profiles, and compared with isolates from patients suffering either pyelonephritis or septicemia. Results revealed that the majority of the isolates belonged to two pathogenic phylogroups, B2 and D. Approximately 67% of the isolates were multidrug resistant (MDR), with 85% producing extended-spectrum beta-lactamase (ESBL) and 6% producing metallo-beta-lactamase (MBL). The *bla*_CTX-M-15_ genotype was observed in at least 70% of the *E. coli* isolates in each category, conferring resistance to an extended range of beta-lactam antibiotics. Whole-genome sequencing and comparative genomics of the ExPEC isolates revealed that two of the four isolates from SSTIs, NA633 and NA643, belong to pandemic sequence type ST131, whereas functional characteristics of three of the ExPEC pathotypes revealed that they had equal capabilities to form biofilm and were resistant to human serum. Overall, the isolates from a variety of ExPEC infections demonstrated similar resistomes and virulomes and did not display any disease-specific functional or genetic coordinates.

## IMPORTANCE

Infections caused by extraintestinal pathogenic *E. coli* (ExPEC) are of global concern as they result in significant costs to health care facilities management. The recent emergence of a multidrug-resistant pandemic clone, *Escherichia coli* ST131, is of primary concern as a global threat. In developing countries, such as India, skin and soft tissue infections (SSTIs) associated with *E. coli* are marginally addressed. In this study, we employed both genomic analysis and phenotypic assays to determine relationships, if any, among the ExPEC pathotypes. Similarity between antibiotic resistance and virulence profiles was observed, ST131 isolates from SSTIs were reported, and genomic similarities among strains isolated from different disease conditions were detected. This study provides functional molecular infection epidemiology insight into SSTI-associated *E. coli* compared with ExPEC pathotypes.

## INTRODUCTION

*Escherichia coli* is one of the most important agents of extraintestinal infections, with the potential to cause infection in almost any anatomical site. It can be grouped into pathotypes, such as uropathogenic *E. coli* (UPEC), septicemia-associated *E. coli* (SePEC), skin and soft tissue infection (SSTI)-associated *E. coli*, neonatal-meningitis-causing *E. coli* (NMEC), and avian-pathogenic *E. coli* (APEC) (1, 2). Urinary tract infections (UTIs) alone account for about 25% of all cases of septic shock, while soft tissue infections represent 10% of reported severe sepsis cases (3, 4). Clinical stages of UTIs vary from nonsymptomatic acute infection to severe septic shock. In the latter case, the infection generally progresses from the urinary tract to the bladder (cystitis) and from the bladder to the kidneys (pyelonephritis) and finally into the bloodstream (urosepsis). Skin and soft tissue infections (SSTIs) are major bacterial infections, often self-limiting but in severe cases requiring hospitalization and parenteral antibiotic therapy. With the indiscriminate use of antibiotics and emergence of multidrug-resistant (MDR) organisms, such as sequence type 131 (ST131) and carbapenem-resistant strains, treatment has become quite challenging (5–7). Despite the fact that SSTI *E. coli* is an important pathotype, to our knowledge only UTI *E. coli* isolates isolated in India have been characterized (8, 9). This study provides a functional molecular infection insight for *E. coli* associated with SSTI. Phylogenetic relationships, virulence profiles, antibiotic resistance patterns, and functional attributes were determined, and whole genomes were sequenced and compared with septicemia and pyelonephritis isolates from the same setting of endemicity in India.

## RESULTS

### Phylogenetic groups and antimicrobial resistance.

The majority of the isolates included in this study were assigned to virulence B2 (36%) and D (29%) phylogroups, with 23% in the A group and 12% in the B1 group (Table 1). Of the three disease types, more than half of the isolates fell into B2 and D phylogroups. In total, 73 isolates (93%) were resistant to at least one of six antimicrobial agents, with highest resistance being shown to ciprofloxacin (86%), followed by tetracycline (77%), co-trimoxazole (72%), gentamicin (31%), chloramphenicol (23%), and fosfomycin (5%). Details of all bacterial isolates, including their antimicrobial resistance patterns, are provided in Table S1 in the supplemental material. Overall, 67% of the total *E. coli* isolates were found to be multidrug resistant (MDR), in which pyelonephritis, septicemia, and SSTI contributed fractions of 0.40, 0.32, and 0.26, respectively (Table 2). The extended-spectrum-beta-lactamase (ESBL)-producing *E. coli* strains were found to comprise as much as 85% of the isolates and were similar in all three disease types. Only 6% of the isolates were metallo-beta-lactamase (MBL) producers. Resistance gene profiling was compatible with the phenotypic observations, with *bla*_CTX-M-15_ being the predominant genotype and NDM-1 being a common occurrence among all MBL producers (Tables 1 and 2). The gene *sul1* was detected in approximately 71% of the isolates in all three categories, conferring resistance to sulfonamides, while the plasmid-based fluoroquinolone resistance gene [*aac(6′)-lb-cr*] was detected in 30% and 71% of the pyelonephritis and SSTI isolates, respectively.

10.1128/mBio.01070-17.1 TABLE S1  Phylogroup, extended-spectrum beta-lactamase (ESBL) production phenotype, and antimicrobial resistance patterns of ExPEC isolates against six non-beta-lactam antibiotics. Download TABLE S1, DOCX file, 0.03 MB. Copyright © 2017 Ranjan et al. 2017 Ranjan et al. This content is distributed under the terms of the Creative Commons Attribution 4.0 International license.

**TABLE 1:** Phylogenetic grouping, virulence, and antimicrobial resistance genotype of extraintestinal pathogenic *E. coli* included in this study^a^

Characteristic	Gene or specific trait	No. (%) of strains associated with infection:		
		Pyelonephritis (*n* = 30)	Septicemia (*n =* 27)	Skin and soft tissue infections (*n* = 21)^b^
Phylogenetic grouping, (no. [%] of strains)				
A (18 [23])		2 (7)	8 (30)	8 (38)
B1 (9 [12])		3 (10)	4 (15)	2 (10)
B2 (28 [36])		15 (50)	7 (26)	6 (29)
D (23 [29])		10 (33)	8 (30)	5 (24)
Virulence factors				
Toxins	*sat*	16 (53)	9 (33)	6 (29)
	*usp*	10 (33)	6 (22)	6 (29)
	*cvaC*	6 (20)	6 (22)	15 (71)*
Adhesins	*fimH*	27 (90)	24 (89)	13 (62)*
	*papC*	28 (93)	25 (93)	5 (24)*
	*afaB/C*	8 (27)	1 (4)	1 (05)
	*sfaD/E*	2 (7)	2 (7)	1 (05)
Protectins	*traT*	5 (17)	4 (15)	6 (29)
	*ibeA*	3 (10)	3 (11)	1 (5)
Iron acquisition	*iroN*	27 (90)	21 (78)	16 (76)
	*iucD*	19 (63)	16 (59)	15 (71)
Antibiotic resistance profiling: antibiotic class				
ESBL	*bla*_CTX-M-15_	21 (70)	24 (89)	17 (81)
	*bla*_TEM_	15 (50)	14 (52)	4 (19)*
	*bla*_CTX-M-15_ + *bla*_TEM_	11 (37)	12 (44)	4 (19)
Metallo-beta-lactamase	*bla*_NDM1_	0 (0)	1 (4)	5 (24)*
Tetracyclines	*tetA*	20 (67)	17 (63)	8 (38)
Aminoglycosides	*strA*	14 (47)	10 (37)	9 (43)
Fluoroquinolone	*aac(6′)-lb-cr*	9 (30)	13 (48)	15 (71)
Sulfonamides	*sul1*	25 (83)	23 (82)	15 (71)
	*sul2*	12 (40)	7 (26)	6 (29)
Trimethoprim	*dhfr*	8 (27)	9 (33)	2 (10)
Integrin	*int1*	18 (60)	15 (56)	13 (62)

a Total number of strains was 78.

b Asterisks denote a *P* value of <0.05 compared to the septicemia group of strains.

**TABLE 2:** Pathotypes and phenotypic drug resistance and multidrug resistance phenotypes

Antimicrobial class or phenotype	Specific drug	% overall prevalence of resistance phenotype in Indian population	Estimated fraction in infection type:		
			Pyelonephritis (*n* = 30)	Septicemia (*n* = 27)	Skin and soft tissue infections (*n* = 21)
Quinolone/fluoroquinolone	Ciprofloxacin	86	0.37	0.34	0.28
Sulfonamide/trimethoprim	Co-trimoxazole	72	0.43	0.36	0.21
Aminoglycosides	Gentamicin	31	0.33	0.41	0.25
Phenicols/phosphonic acid derivatives	Chloramphenicol	23	0.39	0.50	0.11
	Fosfomycin	5	0.00	0.25	0.75
Tetracyclines	Tetracycline	77	0.43	0.35	0.22
ESBL phenotype	Cefotaxime	85	0.36	0.36	0.27
MBL phenotype	Meropenem	06	0.00	0.16	0.83
Multidrug resistance		67	0.40	0.32	0.26

### Virulence genotypes.

Analysis of the virulence genotype *fimH* showed that it was present in ∼90% of the isolates in each category, with the exception of SSTI isolates (62%). Interestingly, *papC* was found to be prevalent in septicemia and pyelonephritis isolates. However, *afaB*/*C* and *sfaD/E* were present in various proportions in the three groups. Of the three toxin types, *cvaC* was detected, to a lesser extent, in pyelonephritis and septicemia isolates. The prevalence of protectin genes *traT* and *ibeA* was the same as that of iron acquisition genes *iroN* and *iucD* in all three groups (Table 1).

### Whole-genome analysis of resistome and virulome.

**FIG 1:**
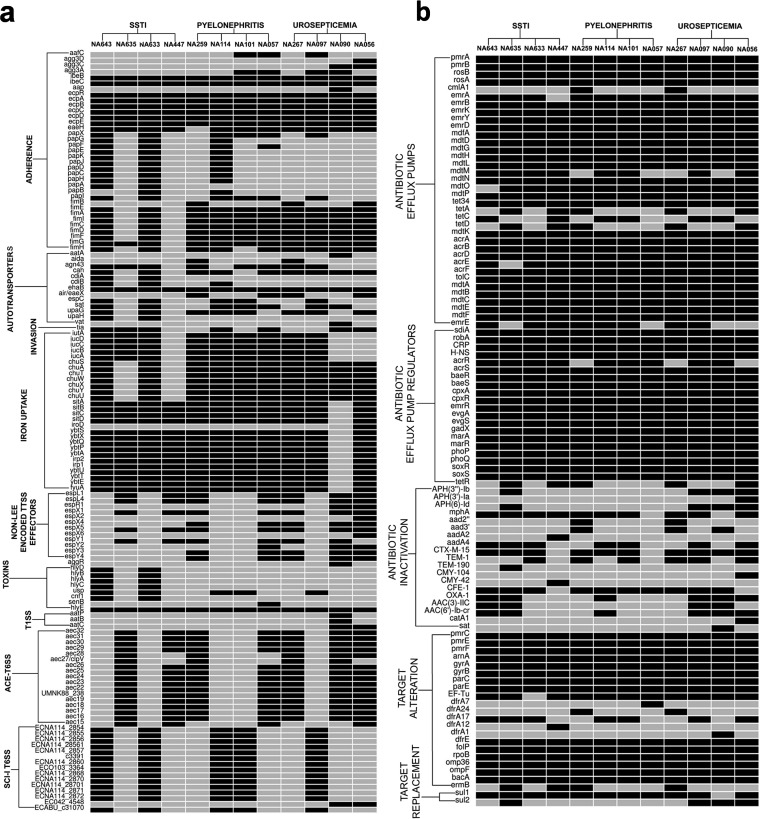
Heat map of virulome (a) and resistome (b) data generated from whole-genome comparative genomics for 12 strains that included four strains from each of the infection categories septicemia, pyelonephritis, and SSTI. Black boxes represent presence, and gray boxes represent absence.

Twelve strains, comprising four from each of the three disease types, were subjected to comparative genomic analysis to obtain a broader perspective of the complexity of the *E. coli* disease types. Genome sequence data for four strains were also included from previous and ongoing studies, including reference strains *Escherichia coli* NA097 and NA114 (10, 11), and eight isolates of this study were subjected to whole-genome sequencing (WGS). Whole-genome-based resistome profiles confirmed the strains to be multidrug resistant. All strains demonstrated relatively similar combinations of resistance genes encoding antibiotic efflux pumps, regulators, antibiotic inactivation, and target modification (Fig. 1b). The virulomes also showed very little pathotype specificity (Fig. 1a). Based on *in silico* multilocus sequence typing (MLST) results, two SSTI strains (NA633 and NA643) were assigned to the pandemic ST131 clone, while the rest of the strains were assigned to different sequence types (STs), namely, ST38, ST68, ST405, and ST 617 (Table 3).

**TABLE 3:** Genome statistics for eight strains sequenced in this study

Strain	Accession no.	No. of raw reads	Genome coverage (fold)	No. of contigs (≥500 bp)	Sequence type	Genome size (bp)	No. of CDS^a^	Coding %	No. of rRNAs	G+C content (%)	*N*_50_ value
NA643	MJDL00000000	2,136,944	108.5	112	ST131	5,322,063	5,257	87.4	17	50.72	216,749
NA635	MJDK00000000	1,822,696	91.8	122	ST617	4,948,859	4,769	87.2	12	50.78	164,858
NA633	MJDJ00000000	1,878,242	94.6	81	ST131	5,275,425	5,212	87.7	17	50.68	265,266
NA447	JWHS00000000	1,500,566	45	239	ST617	5,091,202	4,990	85.7	15	50.68	52,237
NA267	MJDI00000000	2,092,350	105	164	ST405	5,393,308	5,229	86.8	13	50.51	118,812
NA259	MJGD00000000	1,758,550	88.6	162	ST405	5,400,321	5,260	86.9	13	50.51	118,812
NA056	MKHD00000000	1,513,364	78	98	ST68	5,322,471	5,099	87.6	12	50.54	143,398
NA057	JSXL00000000	1,913,156	57	150	ST38	5,286,256	5,173	86.5	8	50.5	120,832

a CDS, coding sequences.

**FIG 2:**
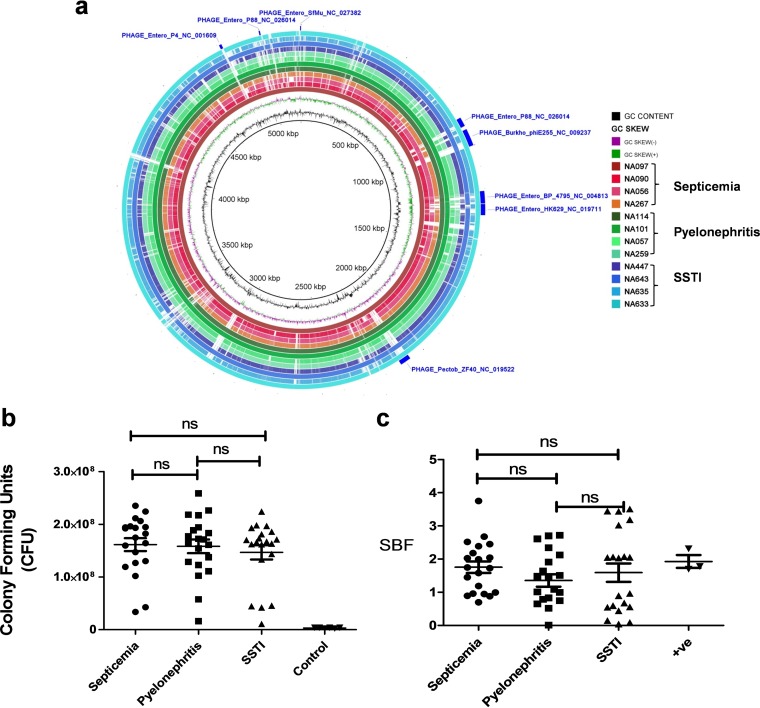
(a) BLAST Ring Image Generator (BRIG) images of 12 strains, including four from each infection category, where each concentric ring represents genomes sequentially in groups, e.g., septicemia, pyelonephritis, and SSTI, generated with the NA097 septicemia strain serving as a reference. (b) Serum resistance tested using 50% human serum. (c) Biofilm formation assay for 10 strains from each category, repeated twice in technical triplicate. ns, nonsignificant; SBF, specific biofilm formation; +ve, positive.

Results of comparative genomic analyses using the BLAST Ring Image Generator (BRIG) platform showed sequence-type-specific similarities, i.e., all five ST131 strains had similar arrangements of genomic regions, whereas isolates belonging to other STs had dissimilar regions (Fig. 2a). Clearly, disease-specific genomic features were not detected.

### Functional virulence determination.

A total of 30 *E. coli* isolates, comprising 10 from each category, were analyzed for functional virulence determinants. Results revealed biofilm formation capabilities for all isolates when tested using M63 medium and incubation for 48 h at 28°C. Most were strong biofilm formers (Fig. 2c) and also able to grow in human serum (50%). Resistance to serum, i.e., demonstrating no deleterious effect, was variable among the isolates, but the difference was insignificant (Fig. 2b).

## DISCUSSION

Infections caused by extraintestinal pathogenic *E. coli* (ExPEC) are common worldwide. In the present study, we observed that *E. coli* strains isolated from SSTI, septicemia, and pyelonephritis were predominately B2 and D phylogroups (Table 1), which are considered virulent *E. coli* (12, 13). The observations of this study are similar to those of Petkovsek et al. (14), who reported that SSTI *E. coli* possessed virulence factors (VFs). Ananias and Yano (15) also showed VFs to be common among different sepsis-associated *E. coli* (SePEC) strains notably able to invade Vero cell lines.

Recent studies reported a global increase in multidrug resistance (MDR) and extended-spectrum beta-lactamase (ESBL) genotypes among ExPEC strains associated with infections (16, 17). In the present study, the majority (67%) of ExPEC isolates were MDR, with CTX-M-15 being the predominant ESBL genotype (Tables 1 and 2). Furthermore, the combination of ESBL and fluoroquinolone resistance was the most frequent, followed by that of CTX-M-15 positivity (CTX-M-15^+^), fluoroquinolone resistance, and co-trimoxazole resistance and that of CTX-M-15^+^, fluoroquinolone resistance, co-trimoxazole resistance, and tetracycline resistance (Table 4). The resistome (Fig. 1b), based on WGS, showed the presence of multiple genes, whereas the MBL phenotype in all isolates was associated with the presence of the NDM gene, reflecting dissemination of NDM carbapenem resistance in India (7).

**TABLE 4:** Prevalence of combinations of ESBL, CTX-M-15, fluoroquinolone resistance, co-trimoxazole resistance, and tetracycline resistance among isolates included in this study (*n* = 78)

Subset	Subset definition^a^	Total no. (%) of Indian isolates	No. of isolates (%) associated with infection type:		
			Pyelonephritis (*n* = 30)	Septicemia (*n* = 27)	Skin and soft tissue infections (*n* = 21)
1	ESBL^+^ CTX-M-15^+^	60 (77)	21 (70)	23 (85)	16 (76)
2	ESBL^+^ CTX-M-15^+^ Tem^+^	30 (38)	11 (37)	12 (43)	7 (33)
3	CTX-M-15^+^, fluoroquinolone resistant	57 (73)	20 (67)	22 (81)	15 (71)
4	CTX-M-15^+^, fluoroquinolone resistant, co-trimoxazole resistant	44 (56)	17 (56)	17 (63)	10 (48)
5	CTX-M-15^+^, fluoroquinolone susceptible, co-trimoxazole resistant	2 (3)	1 (3)	1 (4)	0 (0)
6	CTX-M-15^+^, fluoroquinolone resistant, co-trimoxazole susceptible	13 (17)	3 (10)	5 (18)	5 (24)
7	CTX-M-15^+^, fluoroquinolone susceptible, co-trimoxazole susceptible	3 (4)	0 (0)	1 (4)	2 (10)
8	CTX-M-15^+^, fluoroquinolone resistant, co-trimoxazole resistant, tetracycline resistant	39 (50)	17 (56)	16 (57)	6 (29)
9	CTX-M-15^+^, fluoroquinolone resistant, co-trimoxazole resistant, tetracycline susceptible	4 (5)	0 (0)	0 (0)	4 (19)
10	CTX-M-15^−^, fluoroquinolone susceptible	6 (8)	4 (13)	2 (7)	0 (0)

a Tem, temoniera-beta-lactamase.

ExPEC is known to be evolving and disseminating globally via clonal expansion, with clones of similar genetic architectures but also demonstrating strain-specific features (11, 18). Here, we observed similarities in virulence and resistance coordinates of strains belonging to sequence types (STs) ST131, ST38, ST68, ST405, and ST617. Interestingly, ST-specific commonality but not disease-specific similarity was observed (Fig. 2a). These results support the conclusion that strains of diverse STs are capable of causing similar infections. We also report for the first time the occurrence in India of *E. coli* isolates from SSTIs that carry ST131.

In summary, the results of this study indicate that ExPEC isolates associated with pyelonephritis, septicemia, and SSTIs comprise overlapping phylogroups and patterns of virulence, drug resistance, genomic, and functional properties but are not specifically associated with pathotypes.

## MATERIALS AND METHODS

### Bacterial strains.

A total of 78 isolates, including 21 from patients suffering infections associated with skin and soft tissues, were obtained from the D. Y. Patil Hospital, Pune, India, during 2015. Also, 57 *E. coli* strains isolated during January 2009 to December 2012 were included in the study. All isolates were identified and preserved employing standard laboratory methods of the hospital as previously published (8) and the protocols were approved by Institutional Biosafety Committee (IBSC) of University of Hyderabad, Hyderabad, India.

### Preparation of DNA template and *E. coli* phylogenetic grouping.

Template DNA was prepared using the boiling lysis method. In brief, 100 µl of the bacterial cultures was boiled at 95°C for 20 min and centrifuged at 6,000 rpm for 10 min. The supernatant obtained was used as the template. All *E. coli* isolates were classified into four major phylogenetic groups by multiplex PCR using three molecular markers: *chuA*, *yjaA*, and *tspE4* (19). After electrophoresis (1.5% agarose), gel images were captured and isolates were assigned to phylogenetic groups based on the dichotomous decision tree of the work of Clermont et al. (19).

### ESBL screening and antimicrobial resistance.

ESBL production was confirmed phenotypically, using guideline M31-A3 of the Clinical and Laboratory Standards Institute (CLSI) (20). Resistance to carbapenems was determined by using Etest (HiMedia), and susceptibility was defined by breakpoints per CLSI (20). All *E. coli* isolates were tested for resistance to six major classes of non-β-lactam antibiotics, employing standard disc agar diffusion on Muller-Hinton agar (HiMedia) (20). Six antimicrobial discs (HiMedia) were used: ciproﬂoxacin (30 µg), chloramphenicol (30 µg), fosfomycin (200 µg), gentamicin (10 µg), sulfamethoxazole-trimethoprim (25 µg), and tetracycline (30 µg). Isolates showing resistance to three or more antimicrobial drugs are considered multidrug resistant (MDR).

### Antimicrobial resistance genotyping.

PCR was used to detect β-lactamase genes (*bla*_TEM_, *bla*_SHV_, and *bla*_CTX-M-15_) in ESBL-positive *E. coli* isolates (21, 22). The presence of genes encoding carbapenemase was detected using selected primers (23). Resistance genes {tetracycline resistance [*tetA*], sulfonamide resistance [*sul1* and *sul2*], aminoglycoside resistance [*strA*], the aminoglycoside acetyltransferase [*aac(6′)-lb-cr*], and trimethoprim resistance [*dhfr*]} were determined by PCR using primers and programs reported elsewhere (24, 25). The gene *int1*, encoding class 1 integrase, was also detected by PCR (26).

### Virulence genotyping.

The ExPEC isolates were tested for the presence of 11 *E. coli* virulence genes (VGs) associated with sepsis-related pathophysiology. The genes targeted were of the following four categories: (i) bacterial adhesins (*papC*, *fimH*, *afaB*/*C*, and *sfaD/E*) (27–30), (ii) toxins (*usp*, *sat*, and *cvaC*) (31–33), (iii) protectants (*traT* and *ibeA*) (33, 34), and (iv) the iron acquisition system (*iroN* and *iucD*) (30, 35).

### Whole-genome sequencing and comparative genomics.

Eight of the isolates were sequenced, and comparative genomics analyses were done, including four strains from previous and ongoing studies, providing virulome, resistome, and whole-genome comparisons. Briefly, paired-end sequence data for eight strains were obtained using Illumina MiSeq for the following *in silico* analysis. The NGS QC Toolkit (v2.3.3) (36) was used to filter high-quality reads, followed by contig assembly using SPAdes Genome Assembler (v3.6.1) (37). Numbers of raw reads, respective read lengths, genome coverage obtained after filtering, and total number of contigs were computed. The generated *de novo* contigs were ordered and scaffolded using Contig-Layout-Authenticator (CLA) (38). Final draft genomes were obtained by merging scaffolds using a series of N’s. Draft genomes were submitted to the RAST (39) server for annotation, and genome statistics from the resulting file were extracted using Artemis (40). The sequence type (ST) of each strain was determined by submitting contigs to https://cge.cbs.dtu.dk/services/MLST/.

Twelve strains, including eight from the current study and four from earlier reported and ongoing studies, belonging to different pathotypes were used for the following comparative analysis. BRIG (41) was used to visualize genome variation of the 12 strains. GeneMarkS (42) was used to predict protein sequences. BLASTp (43) analysis of the putative protein sequences was performed against the database of *E. coli* virulence genes downloaded from the Virulence Factors Database (VFDB) (44), providing a virulence profile for each strain. A specific virulence gene was considered present only if the BLASTp hit had identity greater than 60% and query coverage greater than 85%. The presence-absence status of all virulence genes carried by each strain was represented in the form of a heat map using R. A similar heat map was generated for putative resistance-related genes from the Comprehensive Antibiotic Resistance Database (CARD) (45), using BLASTp.

### Phenotypic virulence determination.

Biofilm formation was determined using M63 minimal medium as described earlier (13, 46). Briefly, bacteria grown overnight were diluted to an optical density at 600 nm (OD_600_) of 0.05 in M63 medium, and 200 μl of each was inoculated in triplicate in a 96-well microtiter plate. OD_600_ was measured, and the plate was covered with permeable sealing and incubated at 28°C for 48 h without shaking. Growth was measured after 48 h as OD_600_ using a microtiter plate reader. Medium was removed gently, washed three times with deionized water, and dried. Bacteria were fixed with methanol (99%) for 15 min and stained with 1% crystal violet (30 min) after air drying. Plates were washed three times with deionized water and air dried again. The fixed stained bacteria were resolubilized using 200 μl of ethanol-acetone (80:20), and absorbance was measured at 570 nm. Specific biofilm formation (SBF) was obtained using the formula SBF = (AB − CW)/*G*, where AB is absorbance at 570 nm, CW is OD_570_ of control well (without bacteria), and *G* is growth measured by the formula *G* = OD_600(48 h)_ − OD_600(0 h)_.

Serum bactericidal activity was assayed using 50% human serum as reported previously (13, 46). Briefly, 5 μl of overnight-grown culture was inoculated in 495 μl of fresh LB broth and allowed to grow for 1.5 h at 37°C at 200 rpm. The bacterial cells were pelleted and resuspended in 1 ml of sterile 1× phosphate-buffered saline (PBS). Thirty microliters of bacteria was added to 270 μl of 50% human serum in a 96-well microtiter plate in triplicates, and 30 μl of sample was taken out and plated on LB agar plates after dilution. The plate was covered and allowed to grow for 3 h at 37°C at 100 rpm. After 3 h of incubation, 30 μl of sample was collected again from each well, diluted, and plated on LB agar plates. The bacteria were enumerated after overnight incubation at 37°C, and growth in serum was calculated by subtracting the CFU of 0 h from that of 3 h. A graph was plotted, and results were analyzed. Both of the assays were performed twice and in triplicate on a subset of 10 isolates from each category.

### Statistical analysis.

Statistical analysis of data for virulence and resistance genes was performed employing chi-square and Mann-Whitney tests for serum resistance and biofilm formation, respectively.

### Accession number(s).

GenBank accession numbers of the eight genomes sequenced for this study are MJDL00000000 (NA643), MJDK00000000 (NA635), MJDJ00000000 (NA633), JWHS00000000 (NA447), MJDI00000000 (NA267), MJGD00000000 (NA259), MKHD00000000 (NA056), and JSXL00000000 (NA057). GenBank accession numbers of the other four genomes used are MIPU00000000 (NA114), JSXJ00000000 (NA097), JSXN00000000 (NA101), and MVIO00000000 (NA090).
