# Supplementary material for: Comparative Genomics of Escherichia coli Isolated from Skin and Soft Tissue and Other Extraintestinal Infections
Source: mBio. 2017 Aug 15;8(4):e01070-17. doi: 10.1128/mBio.01070-17 (PMC5559633; doi:10.1128/mBio.01070-17)
Supplement: TABLE S1 [file mbo004173434st1.docx]

| **SI no**  Table ST1: Phylogroup, extended spectrum beta-lactamase (ESBL) production phenotype, and antimicrobial resistance patterns of ExPEC isolates against six non beta-lactam antibiotics. | **Isolate** | **Provisional diagnosis** | **Phylogroup** | **ESBL** | **CIP** | **GEN** | **C** | **COT** | **TE** | **FOS** | **MDR** |
| --- | --- | --- | --- | --- | --- | --- | --- | --- | --- | --- | --- |
|  | **NA001** | PYELONEPHRITIS | B2 | + | R | R | R | R | R | s | Y |
|  | **NA002** | PYELONEPHRITIS | B2 | + | R | S | S | R | R | S | Y |
|  | **NA042** | PYELONEPHRITIS | B2 | + | R | S | S | R | R | S | Y |
|  | **NA072** | PYELONEPHRITIS | B2 | + | R | R | R | R | R | S | Y |
|  | **NA101** | PYELONEPHRITIS | B2 | + | R | R | R | R | R | s | Y |
|  | **NA111** | PYELONEPHRITIS | B2 | + | R | S | S | S | S | s | N |
|  | **NA128** | PYELONEPHRITIS | B2 | + | R | R | S | R | R | S | Y |
|  | **NA140** | PYELONEPHRITIS | B2 | - | S | S | R | R | R | S | Y |
|  | **NA144** | PYELONEPHRITIS | B2 | + | R | R | S | R | R | S | Y |
|  | **NA150** | PYELONEPHRITIS | B2 | - | S | S | S | S | S | S | N |
|  | **NA152** | PYELONEPHRITIS | B2 | + | R | S | S | R | R | S | Y |
|  | **NA259** | PYELONEPHRITIS | B2 | + | R | S | S | R | R | S | Y |
|  | **NA295** | PYELONEPHRITIS | B2 | - | S | S | S | R | S | S | N |
|  | **NA311** | PYELONEPHRITIS | B2 | + | R | S | S | R | R | s | Y |
|  | **NA314** | PYELONEPHRITIS | B2 | + | R | S | S | R | R | S | Y |
|  | **NA046** | PYELONEPHRITIS | D | + | R | S | S | S | R | S | N |
|  | **NA053** | PYELONEPHRITIS | D | - | R | S | S | R | R | S | Y |
|  | **NA057** | PYELONEPHRITIS | D | - | R | S | S | S | S | S | N |
|  | **NA059** | PYELONEPHRITIS | D | + | R | R | S | R | R | S | Y |
|  | **NA064** | PYELONEPHRITIS | D | + | R | S | S | R | R | S | Y |
|  | **NA076** | PYELONEPHRITIS | D | + | S | S | S | R | R | S | N |
|  | **NA080** | PYELONEPHRITIS | D | + | R | S | S | S | R | s | N |
|  | **NA120** | PYELONEPHRITIS | D | - | S | S | S | R | R | S | N |
|  | **NA138** | PYELONEPHRITIS | D | + | R | S | S | R | R | S | Y |
|  | **NA294** | PYELONEPHRITIS | D | + | R | S | R | R | R | s | Y |
|  | **NA116** | PYELONEPHRITIS | B1 | + | R | R | R | R | R | s | Y |
|  | **NA229** | PYELONEPHRITIS | B1 | + | R | R | R | R | R | S | Y |
|  | **NA315** | PYELONEPHRITIS | B1 | + | R | S | S | S | R | S | N |
|  | **NA043** | PYELONEPHRITIS | A | + | R | S | S | R | R | S | Y |
|  | **NA288** | PYELONEPHRITIS | A | + | R | S | S | R | R | S | Y |
|  | **NA035** | SEPTICAEMIA | B2 | + | R | R | R | R | R | S | Y |
|  | **NA040** | SEPTICAEMIA | B2 | + | R | S | S | R | R | s | Y |
|  | **NA099** | SEPTICAEMIA | B2 | + | R | R | R | R | R | S | Y |
|  | **NA100** | SEPTICAEMIA | B2 | + | S | S | S | S | S | S | N |
|  | **NA123** | SEPTICAEMIA | B2 | + | R | R | R | R | R | s | Y |
|  | **NA271** | SEPTICAEMIA | B2 | + | R | R | S | R | R | s | Y |
|  | **NA309** | SEPTICAEMIA | B2 | + | R | S | S | R | S | S | N |
|  | **NA010** | SEPTICAEMIA | D | - | S | S | S | R | S | S | N |
|  | **NA036** | SEPTICAEMIA | D | + | R | R | S | R | R | S | Y |
|  | **NA056** | SEPTICAEMIA | D | + | R | S | R | R | R | S | Y |
|  | **NA090** | SEPTICAEMIA | D | + | R | S | S | R | R | S | Y |
|  | **NA267** | SEPTICAEMIA | D | + | R | R | R | R | R | S | Y |
|  | **NA276** | SEPTICAEMIA | D | + | R | S | S | R | R | S | Y |
|  | **NA279** | SEPTICAEMIA | D | + | R | S | S | R | R | S | Y |
|  | **NA289** | SEPTICAEMIA | D | + | R | S | S | R | R | S | Y |
|  | **NA180** | SEPTICAEMIA | B1 | + | R | S | S | S | R | S | N |
|  | **NA209** | SEPTICAEMIA | B1 | - | S | S | S | S | S | S | N |
|  | **NA230** | SEPTICAEMIA | B1 | + | R | R | R | R | R | S | Y |
|  | **NA287** | SEPTICAEMIA | B1 | + | R | R | R | R | R | R | Y |
|  | **NA018** | SEPTICAEMIA | A | + | S | S | S | R | S | S | N |
|  | **NA212** | SEPTICAEMIA | A | + | R | R | S | R | R | S | Y |
|  | **NA222** | SEPTICAEMIA | A | + | R | S | S | S | R | S | N |
|  | **NA270** | SEPTICAEMIA | A | + | R | R | R | R | R | s | Y |
|  | **NA274** | SEPTICAEMIA | A | - | R | S | R | R | R | S | Y |
|  | **NA317** | SEPTICAEMIA | A | + | R | S | S | S | R | S | N |
|  | **NA318** | SEPTICAEMIA | A | + | R | S | S | S | R | s | N |
|  | **NA320** | SEPTICAEMIA | A | + | R | S | S | S | S | S | N |
|  | **NA602** | SSTI | B1 | + | R | S | R | R | S | S | Y |
|  | **NA604** | SSTI | D | + | R | R | S | R | R | S | Y |
|  | **NA612** | SSTI | A | + | R | S | S | S | R | S | N |
|  | **NA613** | SSTI | A | + | R | S | S | S | R | R | Y |
|  | **NA614** | SSTI | D | - | R | S | S | R | R | S | Y |
|  | **NA618** | SSTI | A | + | R | S | S | S | S | S | N |
|  | **NA629** | SSTI | B1 | + | S | S | S | S | S | S | N |
|  | **NA631** | SSTI | B2 | + | R | R | S | R | S | S | Y |
|  | **NA633** | SSTI | B2 | + | R | S | S | R | S | S | N |
|  | **NA634** | SSTI | D | + | R | R | S | R | S | S | Y |
|  | **NA635** | SSTI | D | + | R | R | S | R | R | S | Y |
|  | **NA637** | SSTI | A | - | R | S | S | S | S | R | N |
|  | **NA638** | SSTI | A | + | R | S | S | R | R | S | Y |
|  | **NA641** | SSTI | D | + | R | S | S | R | R | S | Y |
|  | **NA643** | SSTI | B2 | + | R | S | S | R | R | S | Y |
|  | **NA644** | SSTI | A | + | R | R | S | R | R | S | Y |
|  | **NA651** | SSTI | B2 | + | S | S | S | S | S | S | N |
|  | **NA652** | SSTI | B2 | + | R | S | R | S | R | S | Y |
|  | **NA653** | SSTI | B2 | - | R | S | S | S | R | S | N |
|  | **NA654** | SSTI | B2 | + | R | R | S | S | R | R | Y |
|  | **NA447** | SSTI | A | + | R | S | S | R | R | S | Y |

CIP=Ciprofloaxacin, GEN= Gentamicin, COT= Cotrimoxazole, TE= Tetracycline, FOS= fosphomycin. MDR= multi drug resistance. Y= yes, N= no, R=resistant, S= sensitive, +, positive, -, negative.
